# Resting-state heart rate variability (HRV) mediates the association between perceived chronic stress and ambiguity avoidance

**DOI:** 10.1038/s41598-022-22584-4

**Published:** 2022-10-21

**Authors:** Talita Jiryis, Noa Magal, Eyal Fructher, Uri Hertz, Roee Admon

**Affiliations:** 1 School of Psychological Sciences, University of Haifa, 199 Aba Khoushy Ave. Mount Carmel, 31905 Haifa, Israel; 2 Brus Rappaport Faculty of Medicine, Technion- Israel Institute of Technology, Haifa, Israel; 3 Department of Psychiatry, Rambam Health Care Campus, Haifa, Israel; 4 The Integrated Brain and Behavior Research Center (IBBRC), University of Haifa, Haifa, Israel; 5 Department of Cognitive Sciences, University of Haifa, Haifa, Israel

**Keywords:** Human behaviour, Neurophysiology

## Abstract

Chronic stress is associated with profound behavioral and physiological alterations, including intolerance to uncertainty and reduced resting-state heart-rate-variability (HRV). Critically, uncertainty may arise in situations with known probabilities (risk) or unknown probabilities (ambiguity). Whether associations between chronic stress and decision-making under uncertainty are dependent on the specific type of uncertain decisions, and whether physiological alterations play a role in these putative associations is not yet clear. Here, ninety-two healthy adults that exhibit various levels of perceived chronic stress underwent resting-state HRV recording before completing a behavioral task that involves decision-making under either risk or ambiguity. Computational modelling quantified participants’ behavioral attitudes of approach and avoidance separately for risk and ambiguity. Results indicate, as expected, that perceived chronic stress is positively associated with intolerance to uncertainty and negatively associated with resting-state HRV. Contrary to expectations, behavioral attitudes towards risk and ambiguity were not directly associated with perceived chronic stress, yet HRV fully mediated the association between chronic stress and ambiguity avoidance. Taken together and given the direction of the associations, elevated HRV despite chronic stress may foster adaptive behavior in the form of avoiding ambiguous situations, and hence contribute to reduced exposure to uncertainty and to lower levels of allostatic load.

## Introduction

Encounter with an external or internal demanding stimulus elicits a physiological stress response that involves activation of the sympathetic branch of the autonomic nervous system (ANS)^1–5^. Stress-induced ANS activation immediately leads to an increase in heart rate (HR) and a decrease in heart rate variability (HRV), yielding enhanced physiological arousal towards the challenge. Mutual interactions between the sympathetic and parasympathetic branches of the ANS assure that these acute stress responses are adaptive such that physiological functioning can return to baseline levels upon stress offset. Critically however, in cases where external or internal demands cumulate for a prolonged period of time, the stress response may become chronically active, leading to long-term alterations in physiology and behavior, also known as allostatic load^6–8^. For example, chronically stressed individuals were repeatedly shown to exhibit reduced HRV, including reduced phasic HRV in response to acute stress, and reduced tonic HRV during resting-state^9–14^. Considering that reduced resting-state HRV was documented across multiple chronically stressed cohorts, including chronic workplace stress, social stress and academic stress, it was implicated as a shared physiological marker of chronic stress.

At the behavioral level, chronic stress was associated with intolerance to uncertainty. In fact, uncertainty was recently conceptualized as the essence of stress, such that allostatic load stems from chronic inability to reduce uncertainty^15–17^. Indeed, intolerance to uncertainty, much like chronic stress, has been associated with reduced HRV^18,19^. Critically, individuals vary in the extent to which they interpret and react to uncertainty, with those exhibiting higher levels of intolerance to uncertainty also displaying greater emotional, behavioral and psychophysiological reactivity to uncertain situations, which in turn appears to be a trans-diagnostic risk factor for depression and anxiety^20–23^. To date, only few studies directly assessed the impact of chronic stress on behavior under uncertainty, yielding somewhat mixed findings. For instance, Van Honk et al.^24^ demonstrated that individuals with low baseline cortisol levels display higher rates of risky choices in the Iowa Gambling Task, putatively due to insensitivity to losses and increased reward dependence among these individuals. In another study, hydrocortisone (a corticosteroid) administration was shown to yield increased risk avoidance in a lottery game among male participants^25^. More recently, it was found that chronic stress is associated with decision-making in the context of uncertainty in females but not males^26^.

Importantly, previous decision-making tasks did not consider the differential contribution of risk and ambiguity to uncertainty. To this end, uncertainty may arise in situations where the probability of an outcome is known (risk) but may also arise in situations in which the probability of the outcomes is partially or fully unknown (i.e., ambiguity)^27,28^. For example, if you bet on a specific number and roll a fair dice you know that you have a one in six chance of winning, yet if you hear that the dice that you are about to use will not roll fairly you can no longer accurately predict the likelihood of a specific number, hence the chance of winning becomes ambiguous. While both scenarios involve uncertainty, ambiguous and risky situations were shown to elicit different emotional, neural and physiological responses^29–31^. These differential responses, in turn, may also account for the clear behavioral differences in preference, such that the vast majority of healthy adults would prefer to avoid ambiguous situations compared to risky situations (i.e., ambiguity averseness), suggesting that ambiguity is viewed as more aversive compared to risk^32–36^.

Taken together, independent lines of research explored the associations between chronic stress and reduced HRV, as well as between chronic stress and intolerance to uncertainty. One fundamental open question is whether the association between chronic stress and decision-making under uncertainty is dependent on the specific type of uncertain decisions of risk or ambiguity. A related subsequent question is whether physiological alterations in the form of reduced HRV play a role in these putative associations. In order to address these gaps in the literature, we implemented a decision-making task that involves separate risky and ambiguous trials, and used computational modelling to independently quantify individuals’ behavioral attitudes of approach and avoidance towards risk and ambiguity^30,36,37^. Ninety-two healthy adults with various levels of perceived chronic stress completed this task following a 5-min resting-state HRV recording. We hypothesized that increased levels of chronic stress will be associated with reduced resting-state (tonic) HRV as well as with elevated intolerance to uncertainty, consistent with previous findings. We further hypothesized that associations between chronic stress levels and decision-making under uncertainty will depend on the specific type of uncertain decisions of risk or ambiguity. Finally, we hypothesized that the association between chronic stress and behavioral attitudes of approach and avoidance towards risk and ambiguity will rely on a physiological pathway that is mediated by HRV.

## Materials and methods

### Participants

Ninety-two healthy adult participants were recruited to the study via social media ads (mean age: 25.1, range: 18–43). Participants were predominantly female (68.13%). Participants completed an online screening battery prior to recruitment in order to assess eligibility based on the following exclusion criteria: BMI below 18 or above 30, drug and alcohol abuse, working night shifts, chronic or acute illness, current or chronic use of any medication, current or past neurological or psychiatric disorder including attention deficit hyperactivity disorder (ADHD) for the participants or their first-degree relatives. The experimental protocol was approved by University of Haifa Institutional Review Board (IRB) committee (approval #429/20). All participants were informed about the study procedure prior to signing a written informed consent form. All participants received monetary compensation for their time.

### Procedure

Participants were instructed to refrain from caffeine consumption, eating and physical activity for at least one hour prior to their arrival to the laboratory. Upon arrival, participants signed a consent form and were connected to an Electrocardiography (ECG) device (MindWare mobile). Next, they completed several demographic and psychological questionnaires for approximately 30 min, fostering habituation to laboratory setting. Then, a 5-min resting-state HRV recording was conducted, during which participants were instructed to stay seated and breathe normally. Subsequently, participants performed the risk and ambiguity task as well as another behavioral reversal-learning task unrelated to the current study. Finally, participants were compensated for their time and discharged.

### Measures

#### Perceived stress scale (PSS)

The Perceived Stress Scale (PSS) is a well-established and widely used self-report questionnaire for measuring perceived chronic stress, with high internal reliability (Cronbach’s α > 0.7)^38,39^, that was replicated in the current study (α = 0.89). This 10-item instrument measures the degree to which one’s life situations are evaluated as stressful and the current level of stress experienced. Questions are drafted to point out how respondents perceive their lives as unpredictable, uncontrollable, and overloaded in the past month.

#### Intolerance to uncertainty (IUS-12)

The IUS-12 is a widely used self-report questionnaire that assesses individuals’ reactions to uncertainty, ambiguous situations, and future events^40^. The 12-item questionnaire is a shortened version of the original 27-question Intolerance of Uncertainty Scale^41^, with high internal reliability (Cronbach’s α = 0.91)^40^, that was replicated in the current study (α = 0.74).

#### Risk and ambiguity task

**Figure 1:**
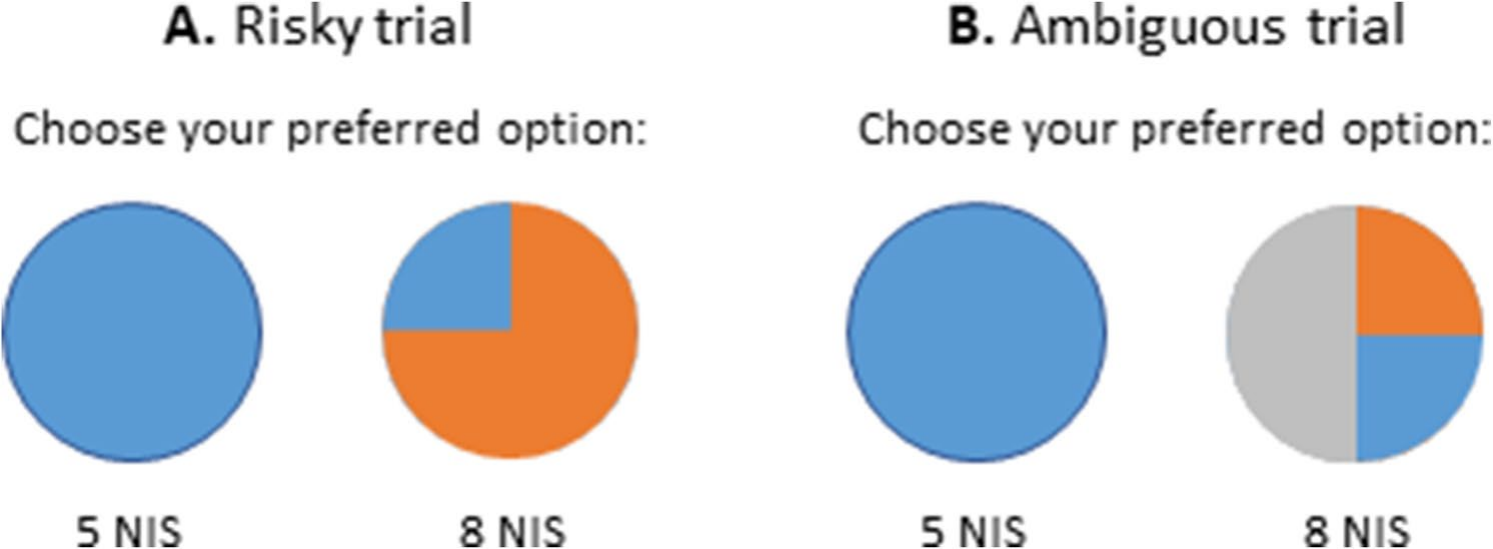
Risk and ambiguity task. Examples of risky (**A**) and ambiguous (**B**) choice trials in the risk and ambiguity task.

The risk and ambiguity task is a behavioral measure for decision making under distinct gamble conditions^30^. The current version of the task included 60 trials during which participants were presented with two options, a safe option for a sure win of 5 NIS or a gamble option. The 60 gamble options varied in A) *Gamble condition* (Risk vs. Ambiguity), B) *Monetary value* (5, 8, 20, 50 or 125 NIS) and C) *Outcome probability* (25%, 50% or 75%). All three conditions in the gamble options pseudo-randomly varied throughout the 60 trials, yielding two repetitions per a specific set of gamble options during the task. In risky trials, the outcome probability values indicated the losing probability if the risk option is selected, whereas in ambiguous trials these values indicated the level of coverage of the outcome probability. Outcome probabilities were depicted as partial coverage area from the entire circle in each trail (Fig. 1). Participants received standardized instructions and the instructor supervised the first few choices to make sure that participants understood the task. Further, participants were informed prior to the task that one of their monetary choices will be randomly selected and given to them upon task completion, in order to create a more realistic and less hypothetical gambling behavior. All participants received additional compensation of 5 NIS upon task completion, representing the selection of a sure win in a given trial. Figure 1A depicts an example of a risky trial in which a participant is presented with a blue circle on the left that represents the sure 5 NIS option vs. a circle on the right that is divided into different outcome probabilities (25%—blue for chance of winning and 75% orange for chance of losing) under various monetary rewards (8 NIS in the example). Figure 1B depicts an example of an ambiguous trial in which the outcome probability is covered by a grey fragment of the circle (50% occlusion in the example). Notably, the objective wining\losing probability in ambiguous trials was always kept at 50%.

#### Heart rate variability (HRV)

Electrocardiography (ECG) signal was acquired using a MindWare Mobile device (MindWare Technologies LTD, Gahanna, OH) at a sampling rate of 500 HZ via three snap electrodes, two on each side of the bottom ribs and one on the right collar bone (clavicle). ECG signal preprocessing was conducted using the python-based package Neurokit2^42^, and included a low pass filter at 5 HZ and a notch filter at 50 HZ. According to published guidelines^43^, R-peaks detection relied on QRS complex that lasted at least 400 ms and with a minimum peak-to-peak interval of 300 ms. Artifacts were detected and corrected based on beat classification^44^. Next, cubic trends were removed from the R-R interval series. The resulting R-R intervals were used for the calculation of HRV time domain measures that have been most typically studied in the context of stress^45^: The root of the mean of the sum of successive differences between adjacent RR intervals (RMSSD) and the standard deviation of the RR intervals (SDNN)^46,47^.

### Statistical analysis

Gambling behavior in the task was first quantified for each participant as the number of gambles taken under each level of risk and ambiguity (i.e., gambling rate). Next, the Gilboa and Schmeidler’s maximum utility model was used to extract behavioral parameters of risk and ambiguity attitudes per participant^37^. This model calculates a subjective value (SV) for each trial while considering the objective winning probability (*p*), the level of ambiguity (A) and the monetary value (v), yielding a risk sensitivity parameter (α) and an ambiguity sensitivity parameter (β). Gilboa and Schmeidler’s maximum utility model equation:

$$SV\left( {p,A,v} \right) = \left( {p - \beta \cdot \frac{A}{2}} \right) \cdot v^{\alpha }$$

In this model, an α < 1 implies risk avoidance while β > 0 implies ambiguity avoidance. The model then assigns a predicted probability of choosing the uncertain option based on its risk, ambiguity, and value, compared with the sure option (fixed 5NIS). Parameters were estimated such that they maximized the model’s predictions’ accuracy, i.e., maximizing likelihood.

$$p\left( {chose\,uncertain} \right) = \frac{1}{{1 + e^{\gamma } \left( {SV_{F} - SV_{v} } \right)}}$$

This was done by a custom code implementing the Gilboa & Schmeidler model in R programming language version 4.0.5^48^ and rstudio version 1.4.1106^49^. All statistical analyses were conducted using SPSS (version 25). Shapiro–Wilk and Kolmogrov-Smirnov tests revealed non-normalized distributions for HRV and behavioral parameters (α & β), hence, log transformed values were used in further analyses. Behavioral parameters under risk and ambiguity were compared using repeated-measures ANOVA. Post hoc comparisons were pursued using Bonferroni correction. Associations between PSS scores, IUS-12 scores, HRV and behavioral parameters were assessed using Pearson’s correlation, with gender and age included as covariates. Finally, a mediation model was tested using AMOS SPSS module (version 20).

### Research involving human participants

All ethical guidelines for human subjects’ research were followed.

## Results

### Behavior under risk and ambiguity

**Figure 2:**
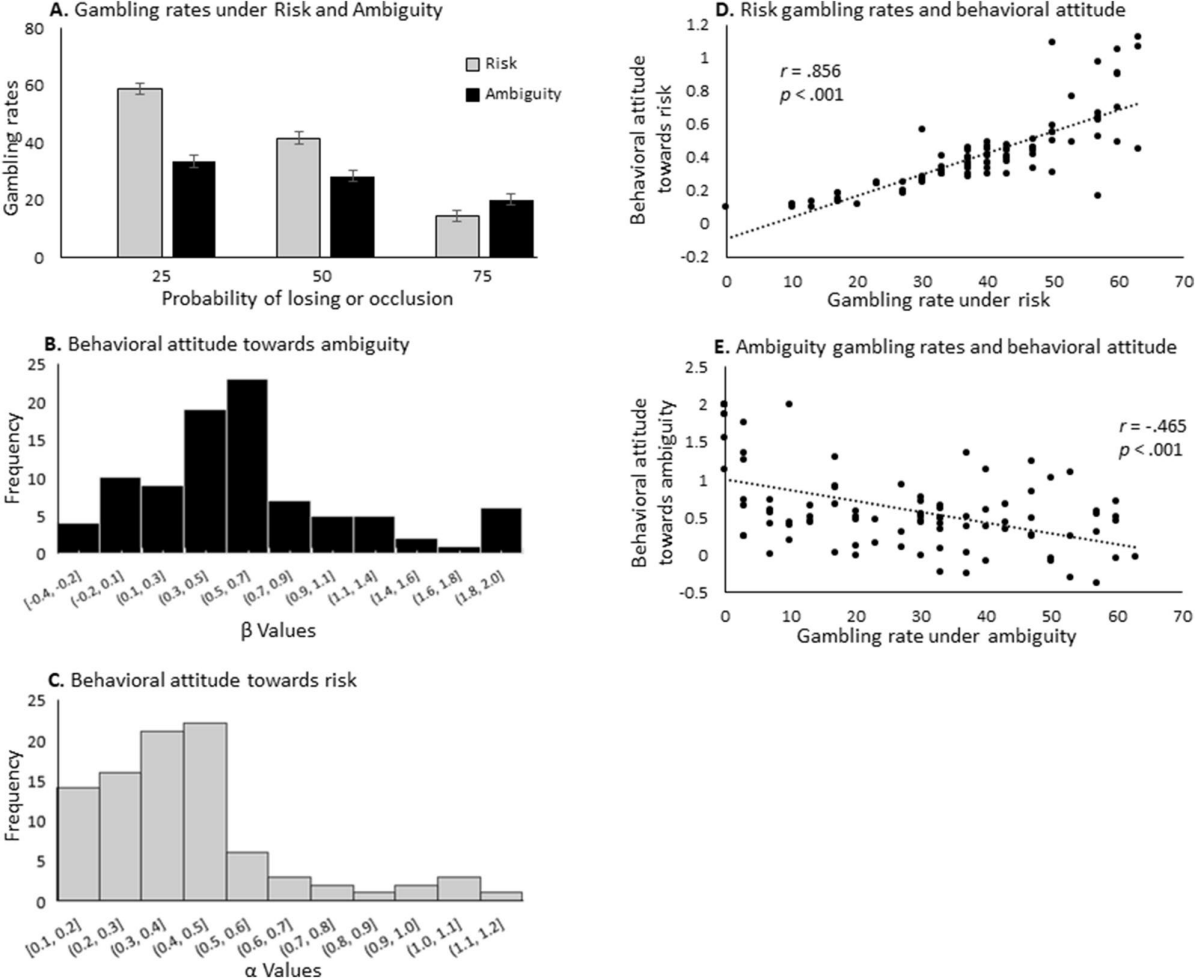
Behavioral attitudes towards risk and ambiguity. (**A**) Gambling rates in ambiguous (black) and risky (gray) trials under three different outcome probabilities. Across the two conditions, lower losing probabilities and lower occlusions were associated with higher gambling rates. (**B** and **C**) Behavioral attitudes towards ambiguity (β) and risk (α) were calculated using a maximum utility model^37^. (**D**) Positive association between gambling rates in risky trials and model-based risk avoidance parameter (α). (**E**) Negative association between gambling rates in ambiguous trials and model-based ambiguity avoidance parameter (β).

Gambling rates in risk and ambiguity trials were assessed using a repeated-measures ANOVA, with *Condition* (Risk vs. Ambiguity) and *Probability* (25%, 50% or 75%, indicating probability of losing or levels of occlusion for risk and ambiguity trials, respectively) as within subject variables. These analyses revealed both a main effect of *Condition* and a main effect of *Probability* (F_(1.86,169.77)_ = 245.82, *p* < 0.001; F_(1,91)_ = 76.24, *p* < 0.001; respectively; Fig. 2A). Mauchly’s test indicated that the assumption of sphericity was violated for the effect of *Probability* (χ^2^_(2)_ = 8.77, *p* = 0.012), in addition, Greenhouse–Geisser’s correction estimates (ε) was > 0.75. Therefore, degrees of freedom were corrected using Hyun-Feldt for the effect of p*robability*. The main effect of *Condition* was driven by higher gambling rates under risk compared to ambiguity (overall gambling rates 38.37% vs. 27.39% for risk and ambiguity, respectively); indicating higher aversion towards ambiguous than risky trials. Notably, in ambiguous trials, under all three occlusion levels, gambling rates remained below 50%. The main effect of *Probability* was driven, as expected, by reduced gambling rates as the probability of losing or of occlusion increases from 25% to 50% to 75% (Post Hoc Bonferroni *p* < 0.001 in all pairwise comparisons). Moreover, results revealed a significant *Condition* by *Probability* interaction (F_(2,182)_ = 62.108, *p* < 0.001). This effect was driven by lower gambling rates under ambiguity compared with risk in trails with 25% and 50% losing or occlusion, and higher gambling rates under ambiguity compared with risk in trails with 75% losing or occlusion (25%: t_(91)_ = 10.84, *p* < 0.001; 50%: t_(91)_ = 7.05, *p* < 0.001; 75%: t_(91)_ = − 3.03, *p* < 0.001, respectively). Repeating the same analysis while controlling for age and gender yielded similar results for *Probability* but not for *Condition* (F_(2,169.29)_ = 10.01, *p* < 0.001; F_(1,88)_ = 2.53, *p* = 0.115, respectively). Further analysis revealed that men gambled significantly more than females under risk (t_(56.16)_ = 2.46, *p* = 0.017) but not under ambiguity (t_(89)_ = 1.2, *p* = 0.23). Finally, across genders, age was not correlated with gambling rates under risk or ambiguity (*r* = 0.139, *p* = 0.190; *r* = 0.066, *p* = 0.533, respectively).

### Computational modelling of behavioral attitudes towards risk and ambiguity

Behavioral attitudes of approach and avoidance towards risk and ambiguity were assessed using a maximum utility model^37^. Figure 2B,C depict the outcome parameters of the model, indicating substantial variability within the cohort in individuals’ attitudes towards risk and ambiguity. Importantly, model parameters outcomes for risk and ambiguity attitudes were not correlated across participants, indicating that these parameters represent independent behavioral attitudes (*r* = 0.047, *p* = 0.659). In order to validate model performance, model parameters were compared with participants’ actual gambling behavior under risk and ambiguity. As can be seen in Fig. 2D,E, the model-independent behavioral measure of average gambling rate across risky trails was positively correlated with model based risk avoidance parameter α (*r* = 0.856, *p* < 0.001), while model-independent average gambling rate across ambiguous trails was negatively correlated with model based ambiguity avoidance parameter β (*r* = − 0.465, *p* < 0.001). This result is in line with the fact that the higher the value of the model derived α parameter the less risk avoidant is the participant, while for ambiguity the opposite is true, with higher model derived β parameters indicating more ambiguity avoidance.

### Perceived chronic stress and intolerance to uncertainty

**Figure 3:**
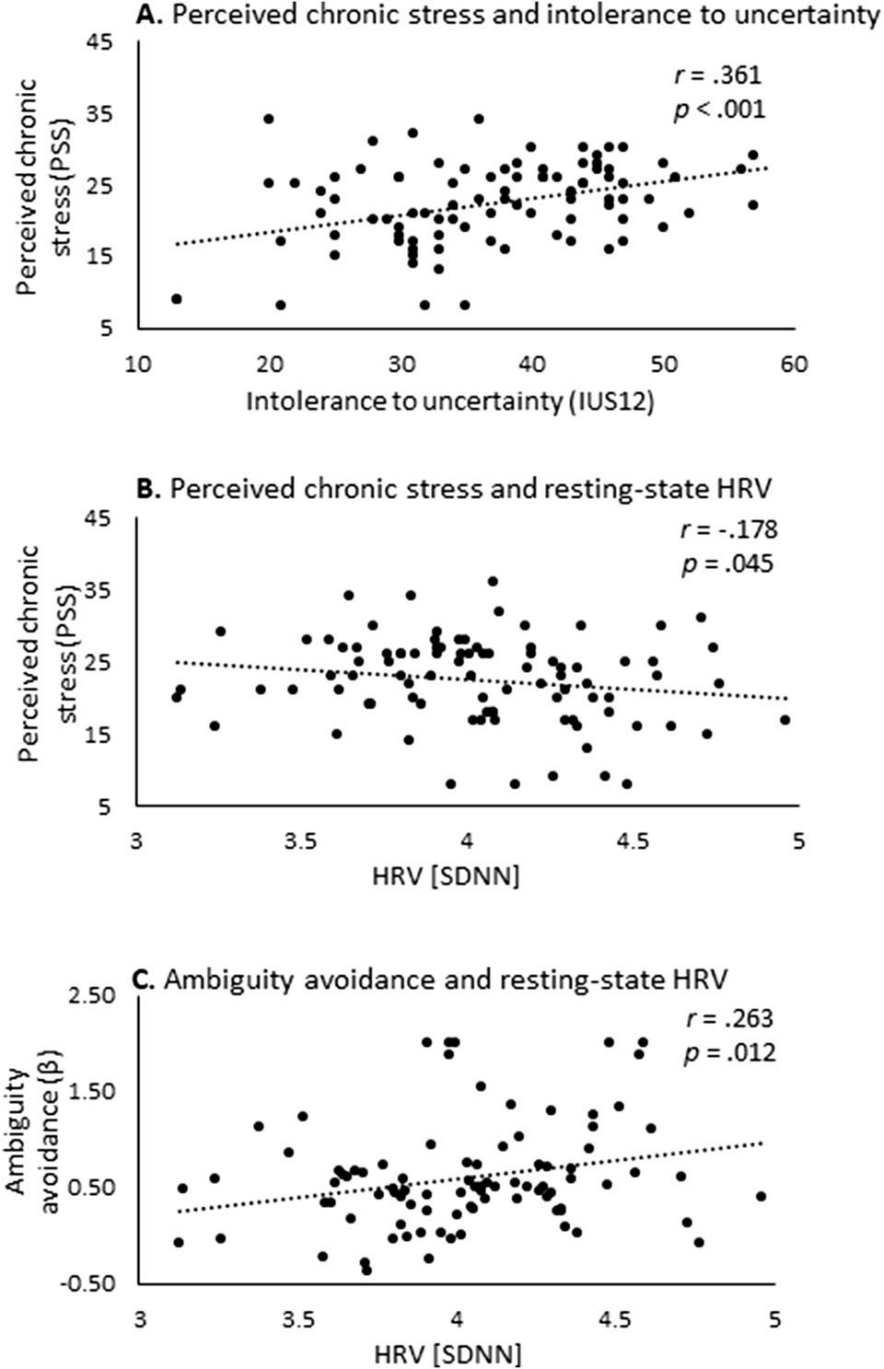
Perceived chronic stress, intolerance to uncertainty, physiology, and behavioral attitudes. (**A**) Positive association between perceived chronic stress and intolerance to uncertainty. (**B**) Negative associations between perceived chronic stress and resting-state HRV [SDNN]. (**C**) Positive association between behavioral attitude towards ambiguity (β) and HRV [SDNN], indicating that ambiguity avoidance is associated with higher resting-state HRV. HRV—Heart rate variability; SDNN—Standard deviation of the RR intervals.

As expected, higher levels of perceived chronic stress were strongly associated with elevated intolerance to uncertainty across participants (*r* = 0.384, *p* < 0.001; Fig. 3A). Repeating the same analysis while controlling for age and gender yielded a similar result (*r* = 0.361, *p* < 0.001).

### Perceived chronic stress, intolerance to uncertainty and behavioral attitudes

Contrary to expectations, variability in perceived chronic stress levels was not correlated with behavioral attitudes towards risk (*r* = − 0.071, *p* = 0.501) or ambiguity (*r* = − 0.020, *p* = 0.850). The same emerged with respect to self-reported intolerance to uncertainty, yielding no significant associations with behavioral attitudes towards risk or ambiguity (*r* = − 0.012, *p* = 0.909; *r* = 0.083, *p* = 0.435, respectively). Repeating the same analysis while controlling for age and gender yielded similar results (Perceived chronic stress*: r* = − 0.139, *p* = 0.204; *r* = 0.017, *p* = 0.870; Intolerance to uncertainty: *r* = 0.020, *p* = 0.855; *r* = 0.030, *p* = 0.777, for risk and ambiguity respectively).

### Perceived chronic stress, intolerance to uncertainty and physiology

As expected, higher levels of perceived chronic stress were associated with lower levels of resting-state HRV. This association emerged with respect to SDNN but not RMSSD (SDNN: *r* = − 0.178, *p* = 0.045; RMSSD: *r* = − 0.152, *p* = 0.075; Fig. 3B). Repeating the same analysis while controlling for age and gender suppressed the significant effect for SDNN (*r* = − 0.159, *p* = 0.132). With respect to intolerance to uncertainty results revealed no significant associations with resting-state HRV (SDNN: *r* = − 0.167, *p* = 0.114; RMSSD: *r* = − 0.108, *p* = 0.308).

### Physiology and behavioral attitudes

Considering its significant association with perceived chronic stress, we further assessed whether SDNN is also associated with behavioral attitudes towards risk and ambiguity (α and β). These analyses revealed a significant association between SDNN and behavioral attitude towards ambiguity (*r* = 0.263, *p* = 0.012) but not towards risk (*r* = 0.048, *p* = 0.326), indicating that ambiguity avoidance is positively associated with resting-state HRV (Fig. 3C). Repeating the same analysis while controlling for age and gender yielded similar results (*r* = 0.246, *p* = 0.019; *r* = 0.745, *p* = 0.458, respectively).

### Perceived chronic stress, physiology, and behavioral attitudes

**Figure 4:**
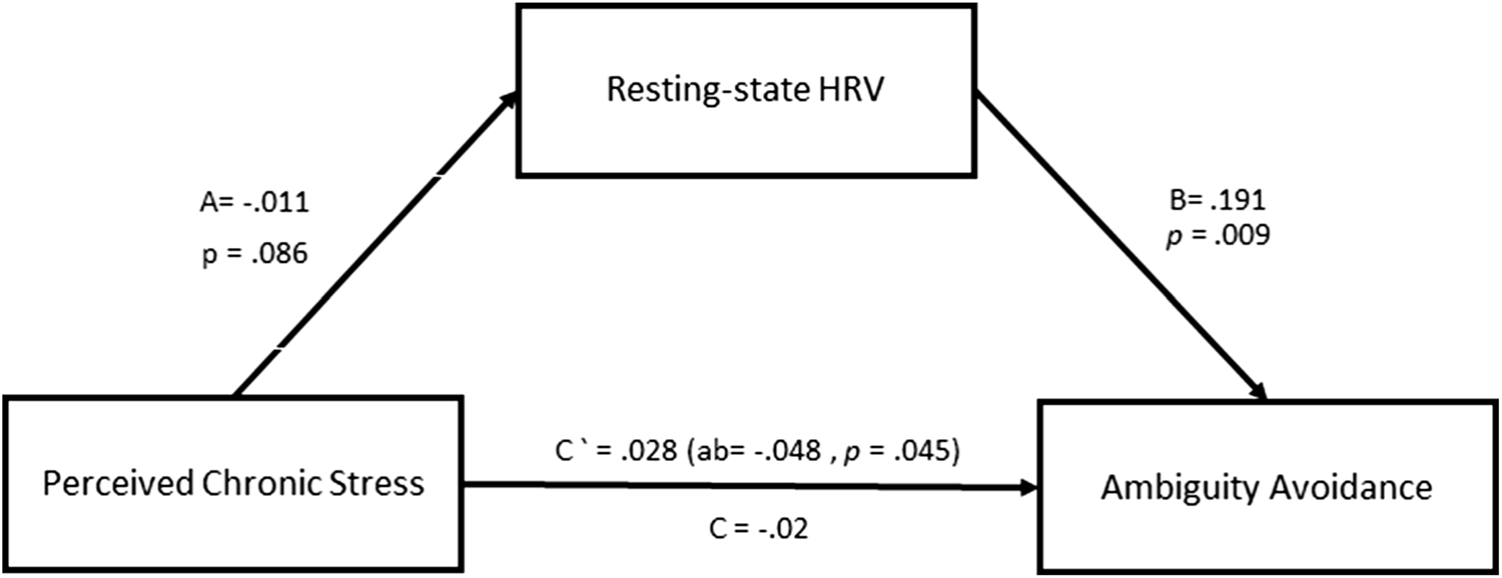
Mediation model. Resting-state HRV fully mediates the association between perceived chronic stress and ambiguity avoidance. (**C**) represents the standardized total direct effect, (**C’**) represents the standardized direct none-mediated effect in the presence of the mediator (HRV) and (**A** and **B**) represent the standardized indirect (mediated) effects.

Since there was no direct association between perceived chronic stress and behavioral attitude towards ambiguity, yet there were significant associations between HRV SDNN and behavioral attitude towards ambiguity as well as perceived chronic stress, we assessed whether HRV SDNN may mediate the putative association between perceived chronic stress and behavioral attitude towards ambiguity (ambiguity avoidance). As depicted in Fig. 4, an indirect model revealed that HRV SDNN indeed fully mediates the association between perceived chronic stress and ambiguity avoidance (AB: − 0.048, CI − 0.128 to − 0.001, *p* = 0.045). This mediation model stems from a direct negative association between perceived chronic stress scores and HRV SDNN (*r* = − 0.178, *p* = 0.045), and a positive direct association between HRV SDNN and ambiguity avoidance (r = 0.263, *p* = 0.012), that remains significant in a multiple regression analysis when perceived chronic stress is included in the model (*Beta* = 0.268, *p* = 0.012). This result depicts a negative indirect-only mediation, since it includes a significant indirect effect without a direct effect, hence, there may be an association between perceived chronic stress and ambiguity avoidance, and if such association exist it is mediated by HRV.

## Discussion

Chronic stress has been associated with profound physiological and behavioral alternations including reduced tonic HRV and elevated intolerance to uncertainty^9–17^. Our results in a sample of healthy adults that exhibit various levels of perceived chronic stress support and replicate these findings. Our results are also supportive of previous findings that suggest that healthy adults typically would prefer to avoid uncertainty when probabilities are unknown (i.e., ambiguity) compared to uncertainty with known probabilities (i.e., risk)^32–36^. Uniquely in here, we assessed whether associations between perceived chronic stress and decision-making under uncertainty are dependent on the specific type of uncertain decisions of risk or ambiguity. Somewhat surprisingly, neither attitudes towards risk nor towards ambiguity were directly associated with levels of perceived chronic stress, nor were these behavioral attitudes associated with variability in intolerance to uncertainty. Attitude towards ambiguity was, nevertheless, positively associated with HRV, indicating that ambiguity avoidance is associated with higher resting-state HRV. Furthermore, the negative association between perceived chronic stress and HRV and the positive association between ambiguity avoidance and HRV together supported a mediation model in which resting-state HRV fully mediates the association between perceived chronic stress and ambiguity avoidance.

Previous studies that demonstrated associations between chronic stress and intolerance to uncertainty led to the conceptualization of uncertainty as the essence of stress^15–17^. This notion is supported by evidence that high intolerance to uncertainty manifests as the interpretation of ambiguous information as threatening, and hence uncertain situations may trigger the stress response, particularly among individuals who are intolerant of uncertainty^20–23^. On the other hand, uncertainty (unpredictability and uncontrollability) is critical for stress response elicitation^50^, and thus intolerance to uncertainty and chronic stress are mutually connected^15–17^. Interestingly however, previous studies that investigated behavioral attitudes towards uncertainty failed to establish consistent relations to chronic stress^27^. For instance, females and males were shown to exhibit different weighting of expected uncertainty under chronic stress^26^. The underlying assumption of the current study was that these inconsistencies may stem from the fact that previous decision-making tasks did not consider the differential contribution of risk and ambiguity to uncertainty. Indeed, an elegant study recently demonstrated that physiological arousal plays a differential role in shaping decision-making under risk vs. under ambiguity^30^. Specifically, the authors found that arousal in the form of skin conductance response decreases risk-taking under risky trials but increases risk-taking under ambiguity. By implementing the same task in here and applying computational modelling to quantify behavioral attitudes of approach and avoidance separately for risk and ambiguity, we hypothesized that risk or ambiguity attitudes will be differentially associated with elevated chronic stress. Results revealed that neither of these behavioral tendencies were directly related to the levels of chronic stress. This surprising result is somewhat in line with a recent study that found no effect of acute stress on behavioral attitudes towards risk and ambiguity^36^. Here, behavioral attitude towards ambiguity (i.e., ambiguity avoidance) was however positively correlated with resting-state HRV. This result, at first, may seem counterintuitive, particularly given that ambiguity avoidance was regarded as a behavioral probe of intolerance to uncertainty^40^, and hence is expected to be negatively correlated with HRV. Indeed, in here, intolerance to uncertainty (IUS-12) was positively associated with chronic stress, which in turn was negatively associated with resting-state HRV.

What then may account for the positive association between behavioral ambiguity avoidance and tonic HRV? First, reduced tonic HRV is considered a prominent physiological marker of chronic stress and allostatic load, that may contribute to impaired decision-making abilities among chronically stressed individuals^51,52^. Second, in light of the strong associations between chronic stress and intolerance to uncertainty, it was suggested that allostatic load may stem from chronic inability to reduce uncertainty^15–17^. Therefore, and in light of current results, we can speculate that among healthy adults that exhibit high levels of chronic stress, through maintaining elevated levels of HRV, these individuals exhibit an adaptive ability to actively deal with uncertain situations by avoiding them. In other words, when levels of chronic stress rise and with them uncertainty, being able to maintain elevated HRV could help to adequately deal with these uncertain situations and hence contribute to lower levels of allostatic load. On the other hand, reduced tonic HRV under chronic stress may hinder the execution of adaptive behavior in the form of ambiguity avoidance, thus leading to encounters with more stressful uncertain situations and elevated allostatic load. This notion is in line with the vast literature on the neurovisceral integration model that highlights high tonic HRV as a proxy for adaptive functioning under stress, as well as for improved cognitive performance, leading to better emotional and physical health. According to this model, the ANS is regulated by multiple cortical and sub-cortical structures that modulate physiological arousal in response to stress, implicating HRV as an index for the capacity of the nervous system to adjust to environmental demands^53–56^. These interpretations nevertheless should be done with great caution, particularly since the current study did not involve follow up assessment, and hence whether avoiding ambiguous situations is indeed an adaptive behavioral approach in the long-term is for future studies to determine.

Several additional limitations that should be acknowledged while interpreting current results include the homogeneous nature of our sample, composed mostly of female participants and with a relatively limited age range of young adulthood. This is important given that all of the measures that were assessed in here were previously shown to be modulated by gender and age, including perceived chronic stress^57,58^, tonic HRV^59–64^ and behavior under uncertainty^26^. In fact, some of our findings in here were suppressed when controlling for the impact of gender and age. Therefore, replications in larger and more diverse gender and age groups are warranted. Equally important would be to conduct this study among individuals that exhibit mental and/or physical illness due to chronic stress. Insights derived from these future studies may provide critical evidence to support or challenge our interpretations. Finally, given that the current task considered decisions only in the gain domain, it would be highly informative to assess the emerging associations also in the context of loss. Particularly since ample evidence suggests that gain and loss elicit differential emotional, cognitive, physiological and neural responses^65–68^.

In conclusion, current results suggest that the associations between chronic stress and behavioral attitudes in the context of ambiguity and risk are not straightforward. Instead, the physiological index of resting-state HRV may mediate the association between chronic stress and ambiguity avoidance. Given the direction of the associations, elevated HRV despite chronic stress may foster adaptive behavior in the form of avoiding ambiguous situations, and hence contribute to reduced exposure to uncertainty and to lower levels of allostatic load.

## Data Availability

The data and modeling code that support the findings of this study are available from the corresponding author [R.A.] upon request.
